# A combinatorial identity for rooted labeled forests

**DOI:** 10.1007/s00010-019-00662-9

**Published:** 2019-07-06

**Authors:** Benjamin Hackl

**Affiliations:** Institut für Mathematik, Alpen-Adria-Universität Klagenfurt, Universitätsstraße 65–67, 9020 Klagenfurt, Austria

**Keywords:** Combinatorial identity, Forest, Set partition, Hurwitz multinomial identity, 05A19, 05C05

## Abstract

In this brief note a straightforward combinatorial proof for an identity directly connecting rooted forests and unordered set partitions is provided. Furthermore, references that put this type of identity in the context of *forest volumes* and *multinomial identities* are given.

## The identity

The aim of this note is to provide an elementary and purely combinatorial proof for an identity stated and proved (via induction) by Dorlas, Rebenko and Savoie in [2].

Let *m* and *p* be positive integers with $p\le m$ and define $\Omega = \{x_1, x_2, \ldots , x_m\}$ to be a set of *m* variables. The combinatorial identity of interest is

1 $$\begin{aligned} \sum _{P \in \Pi _p(\Omega )} \prod _{T\in P} \Big (\sum _{x\in T} x \Big )^{{|}T{|}-1} = \left( {\begin{array}{c}m-1\\ p-1\end{array}}\right) \Big (\sum _{x\in \Omega } x\Big )^{m-p}, \end{aligned}$$

where $\Pi _p(\Omega )$ is the set of all set partitions of $\Omega$ that consist of *p* parts. The identity is particularly remarkable in the sense that it provides a not immediately obvious connection between the “partition world” on the left-hand side and the “binomial world” on the right-hand side.

It should be noted that variations of (1) are well-known and can be found in, e.g., [6, Theorem 5.3.4]. Equations of this type are related to Hurwitz multinomial identities, see [3–5]. In particular, Pitman [4] presents a systematic approach to interpreting identities of this type as decompositions of forest volumes, i.e., polynomials that enumerate special classes of rooted forests. It is not too difficult to apply this framework in order to prove (1)—however, we want to present a more explicit combinatorial proof based on double counting.

It is also easy to see that looking for a combinatorial interpretation in the context of words (i.e., in a setting where the variables in $\Omega$ are non-commutative as the multiplication is replaced by concatenation) is not possible in general, as the following example illustrates.

### Example

Take $m = 5$, $p = 2$ and consider the word $x_1 x_2 x_1$. On the right-hand side the word is constructed once within the parenthesis and then enumerated by the binomial coefficient $$\left( {\begin{array}{c}m-1\\ p-1\end{array}}\right) = 4$$. However, on the left-hand side the word can only be constructed from a single partition part of size 4 containing both $x_1$ and $x_2$, i.e., from the partitions

$$\begin{aligned} \{x_1, x_2, x_3, x_4\}, \{x_5\}, \qquad \{x_1, x_2, x_3, x_5\}, \{x_4\}, \qquad \{x_1, x_2, x_4, x_5\}, \{x_3\}. \end{aligned}$$

Thus, the word $x_{1} x_{2} x_{1}$ occurs only three times on the left-hand side. In general, the left-hand side actually enumerates sets of words over disjoint alphabets, as we take the product over an unordered set partition—in this particular setting, however, the word $x_1 x_2 x_1$ has to be constructed entirely from one of the parts of the partition.

## A refined version and its interpretation

We choose to prove a variation of (1) that belongs to the same family of identities as discussed in [4, Section 4 and Corollary 8 in particular].

### Theorem 1

Let *u* be an additional variable. Then the identity

2 $$\begin{aligned} \sum _{P \in \Pi (\Omega )} \prod _{T\in P} u\Big (\sum _{x\in T} x \Big )^{{|}T{|}-1} = u \Big (u +\sum _{x\in \Omega } x\Big )^{m-1} \end{aligned}$$

holds, where $\Pi (\Omega )$ is the set of set partitions of $\Omega$.

### Remark

Extracting the coefficient of $u^p$ on both sides of (2) immediately yields (1). As we will see in the proof, combinatorially, this identity describes two ways of constructing rooted forests on the vertex set $\{1,2,\ldots ,m\}$: As a set of trees on a partition of the vertex set on the left-hand side, and as the result of deleting the root node in a rooted tree on an extended vertex set on the right-hand side.

### Proof

We claim that both sides of (2) enumerate rooted labeled forests on the vertex set $\{1,2,\ldots , m\}$ with respect to the number of components (enumerated by the variable *u*) and with respect to the out-degree of the vertices (enumerated by the variables $x_j$, respectively).

We begin our proof by observing that as a consequence of the well-known Prüfer bijection (see [1, Theorem 5.19]), the multivariate ordinary generating function

3 $$\begin{aligned} T(u, x_1, x_2, \ldots , x_m)&= (u + x_1 + x_2 + \dots + x_m)^{m-1} u x_1 x_2 \dots x_m \nonumber \\&= \Big (u + \sum _{x\in \Omega } x\Big )^{m-1} u \prod _{x\in \Omega } x \end{aligned}$$

enumerates trees with vertex set $\{0, 1, \ldots , m\}$ where every variable is associated to some vertex (*u* is associated to 0, $x_j$ is associated to *j* for all $1\le j\le m$) and keeps track of the number of vertices adjacent to “their” vertex. In other words, this means that the coefficient of the monomial $u^p x_1^{d_1} x_2^{d_2} \dots x_m^{d_m}$ in $T(u, x_1, x_2, \ldots , x_m)$ is precisely the number of trees with $\deg (0) = p$ and $\deg (j) = d_j$ for all $1\le j\le m$. More details and a proof of this fact are given in, e.g., [1, Theorem 5.19].

We are allowed to think of the trees enumerated by $T(u, x_1, \ldots , x_m)$ as trees rooted at the vertex 0. By removing the product $\prod _{x\in \Omega } x$ from (3) we obtain the right-hand side of our identity (2). Combinatorially, this corresponds to ignoring one neighbor for every vertex except for 0. Equivalently, this can be seen as keeping track of the out-degrees of the vertices instead of their degree. This is because when considering trees rooted at 0, every vertex except 0 has a parent in the tree.

**Fig. 1:**
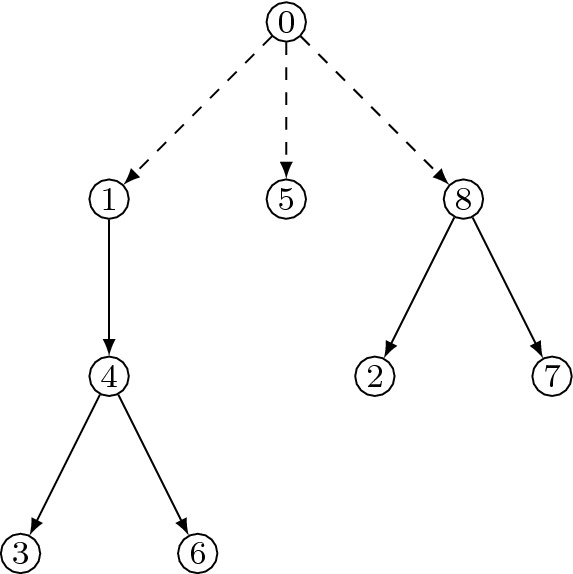
A tree with vertex set $\{0, 1, \ldots , 8\}$ rooted at 0 is in bijection to a rooted forest on $\{1,2,\ldots , 8\}$

Finally, observe that by deleting vertex 0, trees on the vertex set $\{0,1,\ldots ,m\}$ that are rooted at 0 are in bijection to rooted forests on $\{1,2,\ldots ,m\}$ where the out-degrees of the vertices $\{1,2,\ldots ,m\}$ remain unchanged and the number of components in the forest corresponds to the degree of 0. See Fig. 1 for an illustration. This proves our claim for the right-hand side of (2).

The combinatorial interpretation of the left-hand side is quite straightforward. Any rooted forest on $\{1,2,\ldots ,m\}$ can be constructed by starting with an arbitrary set partition $P\in \Pi (\Omega )$, constructing rooted trees whose vertex sets are the subsets of $\{1,2,\ldots , m\}$ corresponding to the partition parts $T\in P$, and combining these trees to a forest.

Obviously, for every set partition *P* the variable *u* keeps track of the number of parts in *P*—and, equivalently, of the number of components of the forest. Finally, note that for ${|}T{|} \ge 2$ we can rewrite

$$\begin{aligned} \Big (\sum _{x\in T} x\Big )^{{|}T{|}-1} = \Big (\sum _{x\in T} x\Big )\Big (\sum _{x\in T} x\Big )^{{|}T{|}-2} = \sum _{r\in T} r\Big (\sum _{x\in T} x\Big )^{{|}T{|}-2}, \end{aligned}$$

which, by the same reasoning as above, enumerates all rooted trees on the vertices corresponding to *T* with respect to the out-degrees of the vertices. The same is true for ${|}T{|} = 1$.

Overall, both sides of (2) can be interpreted as ordinary generating functions for the same family of combinatorial objects—which proves that the identity holds. $\square$
